# The Effect of Enzyme Synergism on Generation of Fermentable Sugars After Alkali Pretreatment of Wheat Straw, Assessed and Predicted Using Multivariate Analysis

**DOI:** 10.3390/polym18020157

**Published:** 2026-01-07

**Authors:** Yufa Gao, Zhe Li, Zhibin Li, Xitao Luo, Mohammad Ali Asadollahi, Safoora Mirmohamadsaghi, Guang Yu, Bin Li

**Affiliations:** 1 National Centre for Archaeology, Beijing 100013, China; 2 Qingdao New Energy Shandong Laboratory, System Integration Engineering Center, Qingdao Institute of Bioenergy and Bioprocess Technology, Chinese Academy of Sciences, Qingdao 266101, China; 3 Weifang Engineering Research Center of Functional Sugar and Polvols, Gaomi Tongli Sugar Co., Ltd., Weifang 261599, China; 4 China National Pulp and Paper Research Institute Co., Ltd., Beijing 100102, China; 5 Department of Biotechnology, Faculty of Biological Science and Technology, University of Isfahan, Isfahan 81746-73441, Iran

**Keywords:** alkali pretreatment, enzymatic hydrolysis, synergistic effect, multivariate analysis

## Abstract

Alkaline pretreatment of wheat straw could significantly augment enzymatic hydrolysis for producing fermentable sugars, which is a pivotal process for the conversion of lignocellulosic biomass into advanced biofuels, biomaterials, or biochemicals. Yet, the enzymatic conversion process system is complex and multivariate, and study on the interaction mechanism of the key parameters in enzymatic hydrolysis is still lacking. Therefore, in this work, multivariate data analysis (MDA) (i.e., principal component analysis (PCA) and partial least square (PLS)) was conducted to reveal the inherent relationship and the significance of these factors in a modified alkali pretreatment system. A robust model, developed from 140 enzymatic hydrolysis datasets, was validated with an additional 20 datasets, demonstrating the predictive prowess of the PLS model. MDA identified that cellulase dosage, mechanical refining, dye adsorption value, and solid content were paramount variables. The integration of cellulase and xylanase notably elevated sugar yields and the conversion rates of carbohydrates, surpassing those of single enzyme treatments. The model’s predictive accuracy, reflected in the close alignment between observed and predicted data, underscores its suitability for optimizing and controlling the enzymatic hydrolysis process. This study paves a way for data-driven strategies to enhance industrial bioprocessing of lignocellulosic feedstocks.

## 1. Introduction

The imperative to discover sustainable energy sources is underscored by the depletion of petroleum-based resources and the concomitant ecological degradation [1]. The conversion of renewable and widely available lignocellulosic biomass (e.g., wood, agricultural wastes, bamboo, crop straw, and energy plants) to advanced biofuels or biomaterials is a viable and attractive way [2,3,4,5]. Lignocellulosic ethanol, a prominent green liquid biofuel, is produced through a biorefinery approach that mainly includes pretreatment, enzymatic hydrolysis, and fermentation [6]. Among them, the hydrolysis of cellulose into sugars for the subsequent fermentation is of crucial importance for the development of the lignocellulosic biofuel industry [7,8,9].

**Figure 1:**
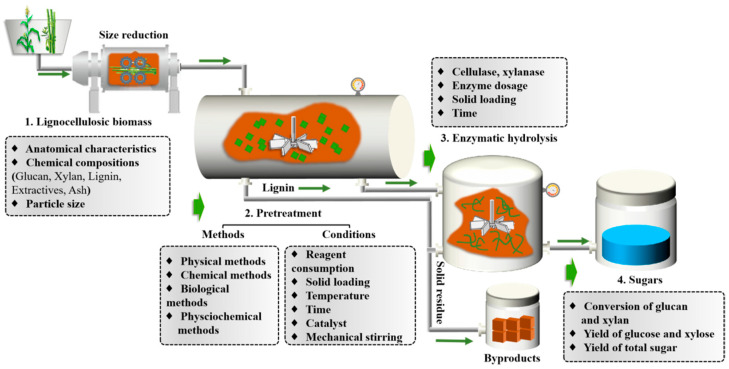
Flow diagram of biomass biorefinery process for conversion of lignocellulose to fermentable sugars, with key parameters of each step.

Pretreatment stands as an essential step for the hydrolysis of cellulosic substrates, as depicted in Figure 1. Most chemical barriers, such as lignin, hemicellulose, and acetyl groups that inhibit the accessibility of enzyme to the cellulose substrate, are removed after pretreatment [10,11,12]. Various pretreatment methods have been developed and employed, mainly including chemical pretreatment [13,14], physical pretreatment [15,16], biological pretreatment [17], and physicochemical pretreatment [18,19]. Therein, alkali pretreatment, as a typical type of chemical pretreatment, is considered as one of the effective and promising methods to promote enzymatic hydrolysis of lignocellulosic biomasses due to its highly efficient and remarkable lignin removal [20,21]. After alkali pretreatment, most hemicelluloses are retained and closely accumulate on the surface of cellulose, which prevents the access of cellulase to the cellulose [22]. A good solution is to use hemicellulase (e. g., xylanase) to hydrolyze hemicellulose and liberate the cellulose [23]. Therefore, it is theoretically useful to achieve higher fermentable sugar yields with a mixture of cellulase and xylanase in enzymatic hydrolysis.

The whole process of converting lignocellulose to fermentable sugars is multivariate, as shown in Figure 1. The features of raw materials (e.g., anatomical characteristics, chemical compositions, and particle size), pretreatment methods and conditions, and enzymatic hydrolysis conditions interact with each other during the whole process of saccharification. The effectiveness of pretreatment under different conditions may have a major impact on enzymatic saccharification, even for the same raw material and pretreatment technology [24,25]. Hence, a comprehensive evaluation of the interaction of the variables in the entire process should be given attention [26,27].

Multivariate data analysis (MDA) is a helpful analytical tool used to analyze or monitor complex operations (such as chemical engineering processes and pulping processes), and it can reveal interactions of the process variables and predict new systems [28,29,30]. Principal component analysis (PCA) and partial least square (PLS) analysis are the commonly used analytical methods for MDA. PCA can reduce a large number of original variables to a smaller number of principal components, which could reflect the maximum amount of variance in the whole dataset. The PLS method can not only reveal the relationships between principal components and regression but also create prediction models that depict the relationship between the two matrices (X and Y) [31]. Both PCA and PLS have been used to analyze variables of pretreatments of lignocellulosic biomass. Baum et al. [32] used MDA to evaluate the pretreatment effects based on the quantitative analysis of NIR spectra of pretreated biomass. Xu et al. [33] analyzed the effects of 10 key factors in the pretreatment and enzymatic processes of saccharification efficiency used by MDA. Our previous work applied MDA to evaluate the alkaline sulfite pretreatment process and PFI refining-assisted alkaline pretreatment of corn stover, and the results showed a good overview of the correlation of factors in the pretreatment system [34,35,36]. However, many studies have applied MDA to the analysis of complex systems with small amounts of data (less than 30 groups). Actually, to obtain valuable analysis results and more reliable predictions of a developed model, the amount of data for reasonable experimental design should be as large as possible. A large amount of experimental data can lead to more accurate MDA analyses and predictive models for better application in complex systems of biomass refining. Also, there are still few reports on the MDA of enzymatic hydrolysis.

Therefore, in this work, to perform the MDA of enzymatic hydrolysis, the lignocellulosic biomass wheat straw was firstly subjected to a modified alkali (NaOH) pretreatment with the supplementation of sodium lignosulfonate (SLS) and anthraquinone (AQ), and then the pretreated wheat straw was enzymatically hydrolyzed to produce fermentable sugars. AQ, as a typical and efficient agent, can promote lignin removal and simultaneously stabilize carbohydrates during pulping or alkali pretreatment with a very small dose of about 0.1% [11,37]. However, AQ is not soluble in water, which could lead to inhomogeneous treatment of substrates. To address this issue, in our previous work, a small amount (2%) of SLS was added during alkali pretreatment, and it was found that SLS as a surfactant could promote the penetration and dispersion of AQ during pretreatment, leading to a 6% improvement in lignin removal and 9% increase in final total sugar yield [11]. Herein, we preliminarily assessed the efficacy of the modified alkali pretreatment and the synergistic interaction between xylanase and cellulase during enzymatic hydrolysis. The novelty of this work lies in the integrated application of multivariate data analysis (MDA) to systematically explore a complex bioconversion process. Specifically, we employed a large, designed dataset to quantify the synergistic interaction between cellulase and xylanase within a modified alkali pretreatment system (enhanced with SLS and AQ). This approach enabled the identification of dominant process variables and the development of a validated predictive model, providing a robust, data-driven framework for optimizing the enzymatic production of fermentable sugars from wheat straw.

## 2. Materials and Methods

### 2.1. Substrate and Chemicals

**Figure 2:**
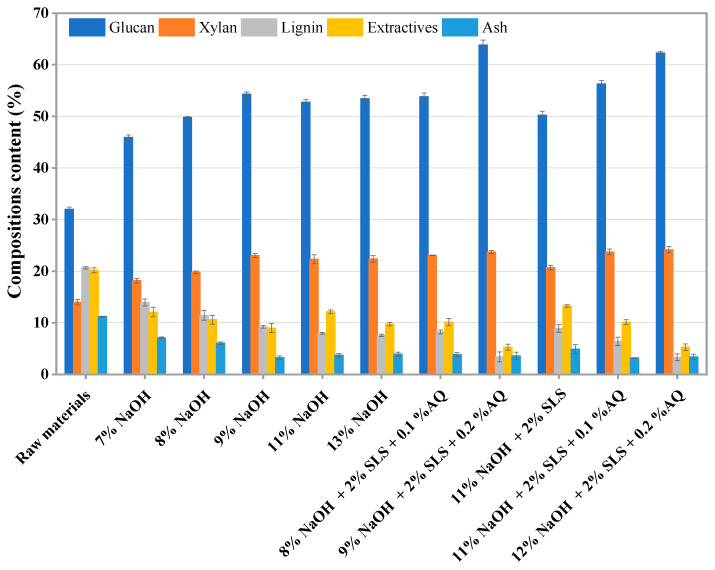
Chemical compositions of the raw and pretreated wheat straw.

Lignocellulosic biomass (wheat straw) used in this study was kindly provided by Dingyuan, Anhui Province, China. Air-dried wheat straw was milled with a planting grinder, then collected and stored in a sealed plastic bag for moisture balance. The chemical compositions of raw and pretreated wheat straw are shown in Figure 2.

Sodium hydroxide, AQ, and SLS were bought from Sinopharm Chemical Reagent Co., Ltd. (Shanghai, China). Enzymes used for enzymatic hydrolysis in this work included commercial cellulase (enzyme activity 80 FPU/mL) and xylanase (enzyme activity 13,200 IU/g), which were obtained from Vland Biotech Inc. (Qingdao, China). All chemicals and enzymes were directly used without further purification.

### 2.2. Modified Alkali Pretreatment of Wheat Straw

The modified alkali pretreatment of the milled wheat straw was performed in a stainless cooking reactor (Mode PL1-00, Xianyang TEST Equipment Co., Ltd., Xianyang, China). There were four cooking tubes in the reactor. For each tube, 50 g (oven-dried) wheat straw was added with the desired alkali pretreatment conditions. Detailed pretreatment conditions are listed in Table S1. The concentration of NaOH was varied within a range of 7% to 13% (*w*/*w*) [11]. This range was selected based on preliminary experiments and reports in the literature, considering both practical effectiveness and economic feasibility. The reactor was heated to the target temperature at a constant rate of 2 °C per minute. Subsequently, the tubes were immediately cooled to below 50 °C by immersion in cold tap water, and then the pretreated wheat straw slurry was transferred into a Nylon bag (300 meshes) and squeezed to separate the solid residue and spent liquor. The solid residue was washed with approximately 5 L of tap water in portions until the filtrate reached a neutral pH. Finally, the washed samples were collected and stored in a freezer (4 °C) for further analyses.

### 2.3. PFI Mechanical Refining

The pretreated wheat straw was refined with a lab-scale PFI mill (PL11-00, Xianyang TEST Equipment Co., Ltd., Xianyang, China). The PFI mill is a typical lab pulp refining instrument that can be used to simulate an industrial disc refiner [20]. The refining consistency of pretreated wheat straw was 10 wt%, and the number of revolutions performed in the PFI refining process were 2000, 4000, 6000, and 8000, respectively. The refining gap was 0.24 mm, and the rotational speed of the PFI refining mill was set at 1460 rpm [20].

### 2.4. Dye Adsorption Measurement

The porosity of wheat straw substrate before and after pretreatment was semi-quantified with the dye adsorption method. The dye applied in this study was Congo red, and the concentration of dye was 5 g/L. In detail, 0.10 g (oven-dried) pretreated wheat straw and 1 mL Congo red were placed into a glass tube (20 mL). Then, the required amount of distilled water was added with a liquor-to-solid ratio of 100:1. The wheat straw was dyed in an incubator at 50 °C with a vibration speed of 90 rpm for 24 h. After dying, the samples were centrifuged at 9000 rpm (4983× *g*) for 5 min to separate the supernatant and substrate. After that, the supernatant was diluted and tested with a UV–Vis spectrophotometer (UV-752, Youke, Shanghai, China) at the wavelength of 492 nm for Congo red. The amount of dye absorbed by wheat straw samples was measured from the decrease in the concentration of the dye solution after dyeing, and it was calculated as mg dye/g wheat straw.

### 2.5. Enzymatic Hydrolysis

The enzymatic hydrolysis experiment was carried out with different solid contents (2–10% (*w*/*w*)) of the pretreated wheat straw in a 40 mL bottle with sodium citrate buffer (0.05 M, pH 4.8) and 0.02% sodium azide according to the National Renewable Energy Laboratory (NREL) standard method (NREL/TP-510-42629, 2008) [38]. The hydrolysis temperature ranged from 40 to 60 °C, and the hydrolysis time varied from 12 to 72 h. An enzyme cocktail of cellulase (0–20 FPU/g substrate) and xylanase (0–80 IU/g substrate) was used in the process of enzymatic hydrolysis. The hydrolysis was performed in an orbital shaker incubator at 150 rpm to ensure consistent mixing and heat transfer. When the enzymatic hydrolysis was finished, the supernatant liquid was divided into two parts. One part of the liquid was filtered with 0.22 μm membrane for monosaccharide analysis, and the other part was further hydrolyzed with a 4 wt% concentration of sulfuric acid to further analyze the total saccharides (including oligosaccharides) that were decomposed and released by the enzymes.

### 2.6. Analytical Procedure for Pretreatment and Enzymatic Hydrolysis Samples

The chemical compositions of wheat straw before and after pretreatment were analyzed according to the NREL procedure. The acidic or enzymatic hydrolysates were tested with high performance liquid chromatography (HPLC) (Model 1200, Agilent, Santa Clara, CA, USA). The HPLC system was equipped with a Bio-Rad Aminex HPX-87H column (300 mm × 7.8 mm) (Agilent, USA).and refractive index detector. The column was run at 55 °C with 5 mM H_2_SO_4_ as mobile phase (0.6 mL/min), and the quantitative analysis was adopted with external standard calibration (sugar samples with known concentrations). All experiments were performed in triplicate. The average values are reported throughout the manuscript, and error bars in the figures represent the standard deviation. The effectiveness of enzymatic hydrolysis was calculated by the following formulas:

*C*-Glucan (%) = (*D*_glucose in hydrolysate_ × 0.9)/*D*_glucan in pretreatment biomass_ × 100% (1)

*C*-Xylan (%) = (*D*_xylose in hydrolysate_ × 0.88)/*D*_xylan in pretreatment biomass_ × 100% (2)

*Y*-Glucose (%) = *D*_glucose in hydrolysate_/*D*_glucose in the corresponding original biomass_ × 100% (3)

*Y*-Xylose (%) = *D*_xylose in hydrolysate_/*D*_xylose in the corresponding original biomass_ × 100% (4)

*Y*-Total sugar (%) = *D*_(glucose + xylose) in hydrolysate_/*D*_(glucose + xylose) in original biomass_ × 100% (5)

Wherein, *C*-Glucan and *C*-Xylan represent the conversion rates of glucan and xylan, respectively. *Y*-Glucose, *Y*-Xylose, and *Y*-Total sugar (*Y*-TS) are the yields of glucose, xylose, and total sugar (glucose + xylose) after enzymatic hydrolysis, respectively. *D* is the dry weight of sugar, 0.9 is the conversion coefficient of glucose to equivalent glucan, and 0.88 is the conversion coefficient of xylose to equivalent xylan.

### 2.7. Calculation of the Degree of Synergism

The degree of synergism (DS) is usually defined as the ratio of the yield of the product obtained when multiple enzymes act simultaneously to the sum of the products obtained when these enzymes act separately, with the same dosage under the same reaction conditions, to the same substrate [39]. The DS can be calculated according to the following equation [40].

*DS* = *C*_1+2_/(*C*_1_ + *C*_2_) (6)

where *C*_1+2_ represents the *C*-Glucan, *C*-Xylan, or *Y*-Total sugar when cellulase and xylanase were added simultaneously, *C*_1_ is the C-Glucan, *C*-Xylan, or *Y*-Total sugar when only cellulase was used, and *C*_2_ is the corresponding *C*-Glucan, *C*-Xylan, or *Y*-Total sugar when only xylanase was added, in enzymatic hydrolysis, to the same substrate.

### 2.8. PCA and PLS Analysis

The data analysis for the PCA model and PLS model was performed with the software of SIMCA-P 11.5 (Soft Independent Modelling of Class Analogy, Umetrics, Umea, Sweden).

## 3. Results

### 3.1. Effectiveness of Alkali Pretreatment

Alkali pretreatment breaks the ether bonds crosslinking lignin and carbohydrates via saponification and solvation. This process may help remove lignin and enhance the accessibility of cellulose to enzymatic action. This process is instrumental in improving the enzymatic saccharification of substrates [41]. The results of pretreatment can be affected by the pretreatment conditions, such as reagent consumption and catalyst. A comparative analysis of the chemical composition of wheat straw before and after alkali pretreatment is presented in Figure 2 and Table S2. The raw material was characterized by a composition of 31.97% glucan, 13.99% xylan, and 20.64% lignin, alongside 20.16% extractives (hot water extractives and ethanol extractives) and 11.23% ash. The alkali-based pretreatment tended to increase the glucan and xylan content relative to the untreated straw. Furthermore, the pretreatment process may contribute to the reduction of extractives, lignin, and ash components originally present in the raw wheat straw. A positive correlation was observed between the concentration of NaOH used in the pretreatment and the extent of lignin removal (Figure 2). Specifically, at an 8% NaOH pretreatment condition, the residual lignin content was reduced to 11.43%. Also, increasing the NaOH concentration to 13% further diminished the lignin content to 7.57%, underscoring the significance of NaOH dosage in optimizing the pretreatment outcome. The compositional changes induced by pretreatment—specifically, lignin removal and carbohydrate retention—directly alter the microstructure of the substrate. The reduction in lignin, a hydrophobic and recalcitrant polymer, diminishes physical barriers and reduces potential sites for non-productive enzyme binding. Consequently, this enhances the accessibility of the cellulose microfibrils to hydrolytic enzymes, which is a prerequisite for efficient saccharification.

The integration of appropriate adjuvants within the alkali-based pretreatment process is essential for enhancing carbohydrate stability and promoting lignin removal. Surfactants, characterized by their hydrophilic and hydrophobic properties, may facilitate the penetration and solubility of lignin during alkali pretreatment [42]. SLS is an effective surfactant. By reducing the surface tension at liquid interfaces, it may accelerate the extraction of hydrophobic components and induce structural alterations within the biomass [43]. Additionally, AQ, through its oxidative capacity on carbohydrate end groups, mitigates hemicellulose degradation via peeling reactions, thus stabilizing the carbohydrates by converting aldehyde groups to carboxyl groups [44,45]. The incorporation of AQ as a catalyst in alkaline pretreatment has been demonstrated to enhance the pretreatment’s effectiveness. In this work, the commercial SLS and AQ were used in alkali pretreatment and modified the pretreatment’s effectiveness. The influence of AQ and SLS on the chemical compositions of wheat straw after pretreatment is shown in Figure 2. It was observed that the addition of these adjuvants results in a clear increase in glucan and xylan contents, while concurrently reducing the lignin content. Specifically, the introduction of 2% SLS and 0.1% AQ during the pretreatment process with 8% NaOH led to a roughly 4% increase in glucan and xylan contents and a 3.2% decrease in lignin content. This enhancement in pretreatment effectiveness is likely associated with the facilitation of lignin removal and the stabilization of carbohydrates by AQ, as evidenced by the oxidation of reducing end aldehyde groups to form stable carboxyl groups [11].

### 3.2. Hydrolysis of Wheat Straw with Cellulase and Xylanase

**Figure 3:**
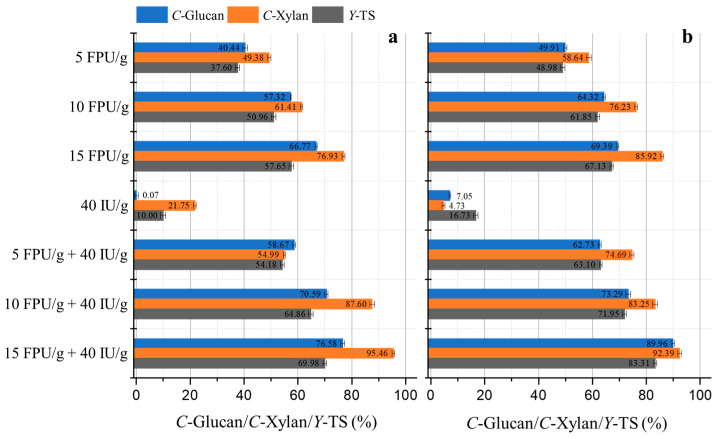
Enzymatic hydrolysis of wheat straw with different ratios of cellulase (FPU/g-glucan) and xylanase (IU/g-glucan) after pretreated with 7% NaOH (**a**) and 11% NaOH (**b**).

After pretreatment, the fine-tuning of enzymatic digestion parameters is identified as a critical step that can further augment the efficiency of enzymatic saccharification. In this work, a variable examination of the combination of both enzymes and their concentrations was executed to delineate the impacts on the saccharification process. Selection of the substrate derived from 7% NaOH and 11% NaOH pretreatments for enzymatic hydrolysis was predicated on a balance between the effectiveness of pretreatment and considerations of economic viability. The saccharification efficacy was gauged based on the conversion rates of glucan and xylan, as well as the overall total sugar yield, serving as the important evaluative metrics. As exhibited in Figure 3, a synergistic application of cellulase and xylanase yielded superior conversion rates for glucan and xylan, as well as a more substantial total sugar yield, surpassing the results of treatments employing either enzyme in isolation.

Illustratively, wheat straw subjected to 7% NaOH pretreatment demonstrated conversion rates of glucan and xylan at 70.59% and 87.60%, respectively, with a total sugar yield reaching 64.86% following a 48-h incubation period with an enzyme cocktail comprising 10 FPU/g of cellulase and 40 IU/g of xylanase. Enzymolysis conducted solely with 10 FPU/g of cellulase yielded markedly lower figures, recording 57.32% for glucan, 61.41% for xylan, and a total sugar yield of only 50.96%. Furthermore, hydrolysis reliant solely on xylanase at a concentration of 40 IU/g yielded minimal conversion rates of 0.07% for glucan, 21.75% for xylan, and a total sugar yield of merely 10.00%. These findings underscore the potential of cellulase, when conjointly applied with xylanase, to improve the hydrolytic efficiency of the substrate under the tested conditions.

The conversion rates of glucan and xylan, as well as the total sugar yield, were found to be lower following the 7% NaOH pretreatment in comparison to the 11% NaOH pretreatment (Figure 3). This disparity may be primarily associated with the higher lignin content in the substrate post 7% NaOH pretreatment (Figure 2). Lignin tends to exhibit a greater affinity for enzyme adsorption than carbohydrate substrates. This non-productive adsorption may reduce the potential efficacy of enzymatic hydrolysis.

Furthermore, the DS is a critical parameter for evaluating the synergistic effect of cellulase and xylanase on the degradation of wheat straw. A DS value exceeding 1.0 indicates a synergistic enhancement, whereas a value below 1.0 suggests competitive interactions between cellulase and xylanase. Table 1 illustrates the changes in DS values between cellulase and xylanase. In this study, an enzyme cocktail with a ratio of 5 FPU/g of cellulase to 40 IU/g of xylanase demonstrated DS values of 1.45 and 1.26 for *C*-Glucan following 7% and 11% NaOH pretreatments, respectively. These values highlighted a significant synergistic effect. This synergism was further supported by the DS values for *C*-Xylan and *Y*-Total sugar, which also exceeded 1.0.

However, an increase in the concentration of cellulase did not yield the expected positive synergistic effect. Specifically, the DS values for *C*-Glucan of wheat straw pretreated with 7% NaOH were 1.45, 1.23, and 1.15 for enzyme combinations of 5 FPU/g + 40 IU/g, 10 FPU/g + 40 IU/g, and 15 FPU/g + 40 IU/g, respectively. Notably, with an enzyme cocktail of 15 FPU/g cellulase + 40 IU/g xylanase, the DS values for *C*-Xylan were observed to be less than 1, with values of 0.93 for 7% NaOH and 0.90 for 11% NaOH, suggesting a competitive interaction between cellulase and xylanase during hemicellulose hydrolysis. The findings underscored the potential for higher enzymatic hydrolysis yields when cellulase and xylanase were used in combination. Nonetheless, excessive enzyme concentrations may lead to enzyme crowding or competition, potentially hindering enzyme adsorption to cellulose and limiting sugar release. These results were in line with the understanding that while synergistic enzyme combinations can enhance the degradation of lignocellulosic biomass, the balance and proportions of enzyme cocktail components are of pivotal importance for optimizing the saccharification process.

### 3.3. PCA Analysis of the Whole Process

The conversion from carbohydrates to sugars is also a multivariate system, and all variables in the system have influence on each other, as shown in Figure 2 and Figure 3. Therefore, the interactions among the multiple variables throughout the system are important to understand and optimize the whole process. In this work, the wheat straw samples were hydrolyzed with enzyme after alkali pretreatment. We analyzed 18 key variables throughout the enzymatic hydrolysis process using PCA and PLS methodologies. This approach allowed for a more profound dissection of their interactions. The 18 key variables were categorized into three groups: Property variables of pretreated wheat straw: glucan, xylan, lignin, extractives, ash, and dye adsorption values (DAV, reflecting substrate porosity); Enzymatic hydrolysis parameters: PFI refining, enzyme dosage (cellulase and xylanase), solid content, pH, hydrolysis duration, and temperature; Enzymatic effect evaluation variables: glucan conversion rate (C-Glucan), xylan conversion rate (C-Xylan), glucose yield (Y-Glucose), xylose yield (Y-Xylose), and total sugar yield (Y-TS). A total of 160 sets of enzymatic hydrolysis data were acquired. Among these, 140 groups were used under different conditions to build the model (for calibration), and another 20 separate groups were employed to tentatively evaluate the predictive capabilities of the built PLS model. The variables were separated into three categories, presented in Table S3, and all data were analyzed with SIMCA-P software 11.5.

**Figure 4:**
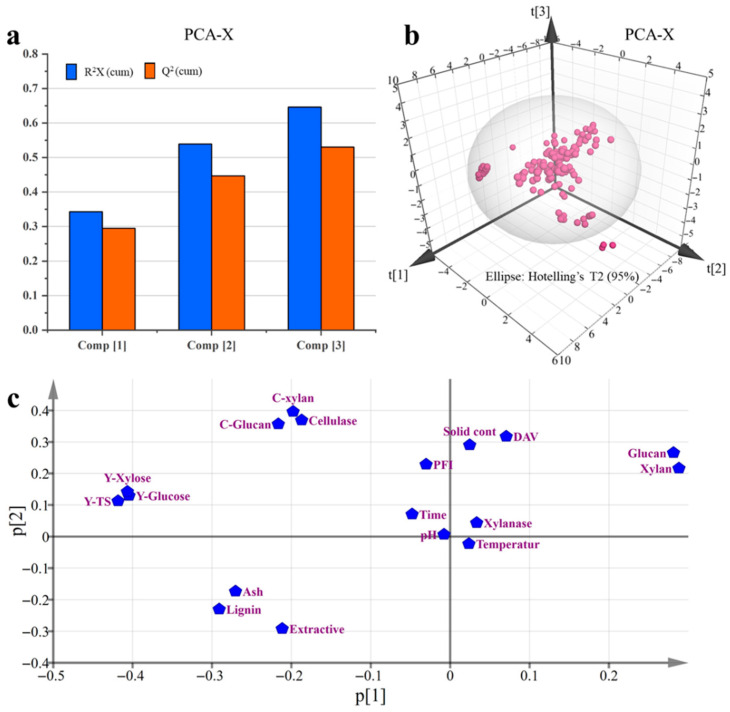
The values of cumulated R^2^ and Q^2^ (**a**), the score plot (**b**) and loading scatter plot (**c**) of PCA analysis.

Before PLS analysis, PCA is usually used to analyze the entire dataset to obtain an overall picture and remove the outliers and variables that are basically constant. As shown in Figure 4a and Table S4, the PCA results showed three principal components, with R^2^X (cum) and Q^2^ (cum) values of 0.646 and 0.530, respectively. The R^2^ value signifies the proportion of variance captured by the model for all variables, where a higher R^2^ indicates a more robust multivariate analysis model. The Q^2^ value is calculated with the cross-validation method, and it is an estimate of the predictive abilities of the PCA model. The Q^2^ value exceeding 0.5 suggests that the PCA model possesses enhanced predictive capabilities for new datasets [33]. Consequently, this PCA model had the role of explaining important valuable information throughout the whole process and showed good predictive ability in this work. Figure 4b presents the score plot for the 140 experimental groups, with the 95% confidence ellipse encapsulating the majority of observations. The presence of a few data points outside this ellipse indicates their classification as outliers within the PCA model framework, and they should be excluded in the following PLS model.

The loading scatter plots comprise a valuable tool for analyzing the intrinsic relationships among variables within the PCA model (Figure 4c). In this model, variables that are close to each other have a direct and strong positive intra-relative relationship. The variables that are positioned opposite to each other are negatively correlated. The observation that *C*-Glucan, *C*-Xylan, and cellulase cluster together suggested a significant positive interrelation among them. On the other hand, lignin, ash, and extractives, known to impede enzymatic hydrolysis, exhibited a negative correlation with *C*-Glucan, *C*-Xylan, cellulase, *Y*-Glucose, *Y*-Xylose, and *Y*-TS. Additionally, the grouped variables (Glucan and Xylan) were located far away from *Y*-Glucose, *Y*-Xylose, and *Y*-TS. This result indicated that the glucan and xylan contents in the pretreated substrate were not positively correlated with the sugar yield, and thus the conditions of the enzyme digestion process had a major impact on the sugar yield, which was a critical part of the whole process.

### 3.4. The PLS Model Analysis for the Whole Process

**Figure 5:**
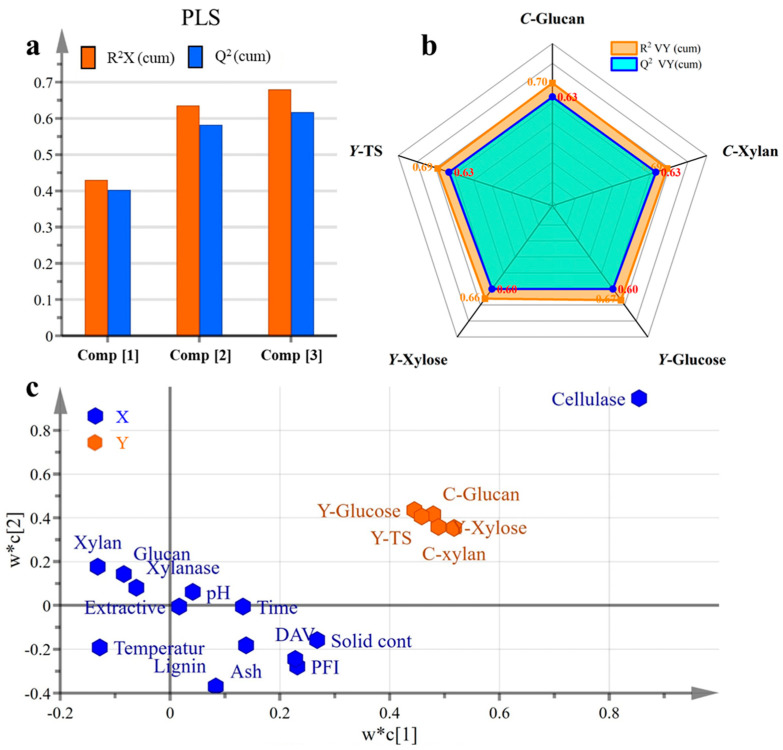
Values of cumulated R^2^ and Q^2^ of principal components (**a**) and Y variables (**b**), and loading scatter plot (**c**) of the PLS analysis.

In the PLS model, the property parameters (including Glucan, Xylan, Lignin, Extractives, Ash, and DAV) of the pretreated wheat straw and enzymatic hydrolysis conditions (i.e., PFI, Cellulase, Xylanase, solid content, Time and Temperature) were set as X variables. The effect variables of enzymatic hydrolysis (*C*-Glucan, *C*-Xylan, *Y*-Glucose, *Y*-Xylose, and Y-TS) were set as Y variables. The three principal components of the PLS model were built. As displayed in Figure 5a and Table S5, the R^2^X (cum), R^2^Y (cum), and Q^2^ (cum) were 0.568, 0.680, and 0.788, which showed that the PLS model had good performance (the PLS model could explain 56.8% of the variation in X variables and 68% of the variation in Y variables, and it had good overall predictability, with a high Q^2^ (cum) > 0.5). Moreover, Figure 5b is a Y overview plot, and it displays the cumulated values of R^2^ and Q^2^ for each Y variable (*C*-Glucan, *C*-Xylan, *Y*-Glucose, *Y*-Xylose, and *Y*-TS). The bars of Q^2^ were all higher than 0.5, indicating that the model was well fit for this enzymatic hydrolysis process and it had good prediction ability. The loading scatter plot suggests that cellulase may be the most influential variable among the parameters examined (Figure 5c). Illustratively, the conversion rates of glucan and xylan, along with the yield of total sugar, achieved respective heights of 77.84%, 89.86%, and 69.89% under the conditions of 11% NaOH pretreatment and an application of 15 FPU/g of cellulase in the following enzymatic hydrolysis. These figures notably surpass the results obtained with an enzyme application of 5 FPU/g. This comparison brings into focus the significance of the quantity of cellulase, thus establishing it as a critical parameter that necessitates careful consideration.

In the PLS model, the variable importance plot (VIP) and coefficient plot are important results that provide insights into the influence of predictors. The VIP is particularly instrumental in assessing the significance of each X variable on the Y variables, with a VIP value exceeding 1.0 denoting important influence [35]. Figure S1a is the corresponding VIP plot for the PLS model, which identified four parameters of notable importance: cellulase (2.61), PFI (1.07), DAV (1.02), and solid content (1.00). Among these, cellulase was the most influential variable within the system, a finding corroborated by the aforementioned results. The PFI and DAV were also important variables. The importance of DAV and PFI refining in the model underscores the critical role of substrate microstructure. A higher DAV indicates a more porous structure that facilitates deeper enzyme penetration [11]. PFI refining is a mechanical treatment that physically shears and fibrillates the biomass, reducing particle size, disrupting the ordered cellulose crystallinity, and significantly increasing the specific surface area [46]. Both of these microstructural modifications—increased porosity and surface area—directly enhance the accessibility of cellulose to enzymes, which is a fundamental driver of the macroscopic hydrolysis rate and final sugar yield.

Additionally, solid content was another key influencing factor in the enzymatic hydrolysis process. Compared with the conversions performed at low solid content, enzymatic hydrolysis performed at high solid content possibly presents many advantages, such as higher sugar concentration and decreased capital and operating costs [46]. However, enzymatic hydrolysis at high solid content faces several limitations [47]. These limits include insufficient available water, difficulties in mixing and handling, inadequate heat transfer, and elevated inhibitor concentrations. For instance, the conversion rates of glucan for a sample pretreated with 11% NaOH were 70.83% at 2% solid content and 48.79% at 10% solid content under identical enzymatic hydrolysis conditions, as depicted in Figure 2. This comparison underscores the important role of solid content in the enzymatic hydrolysis process.

The coefficient plots for all the Y variables (*C*-Glucan, *C*-xylan, *Y*-glucose, *Y*-xylose, *Y*-Total sugar) are shown in Figure S1b–f. Cellulase still had a more positive role in the enzymatic saccharification process than other variables. Notably, ash was an important variable in enzymatic hydrolysis. The ash components in wheat straw, comprising elements such as potassium (K), calcium (Ca), iron (Fe), copper (Cu), magnesium (Mg), silicon (Si), and phosphorus (P), existed in ionic forms. In the enzymatic hydrolysis process, these cations could dissolve into the solution and affect the cellulase activity. Some of these elements are known to modulate enzyme activity; for instance, K^+^ and Mg^2+^ can influence cellulase action, while others like Fe^3+^ and Zn^2+^ can take part in the enzymatic reaction as components. Chen et al. [8] documented that the common cations (such as K^+^, Mg^2+^, and Ca^2+^) of ash had inhibitory effects on cellulase, and Ca^2+^ and Mg^2+^ showed stimulative effects on β-glucosidase. Furthermore, the plots indicate that lignin and temperature play more negative roles in the process. Many references have reported that there may be a strong negative correlation between lignin content and the yield of enzymatic hydrolysis under certain conditions [38]. Enzymes, including cellulase, exhibit optimal function within a specific temperature range, with activity plummeting outside this range. Both excessively high and low temperatures were detrimental to enzymatic processes [48]. According to the above results, pretreatment and enzymatic conditions need to be carefully considered to achieve favorable glucan and xylan conversion and satisfactory total sugar yields.

In general, the total sugar yield of approximately 70% obtained under optimal conditions (11% NaOH pretreatment, 15 FPU/g cellulase) is comparable to or exceeds yields reported for wheat straw pretreated with conventional alkali methods (often ranging from 50 to 65%) [13,21,37]. Furthermore, the predictive accuracy of our PLS model (R^2^ > 0.62 for sugar yields) is consistent with the performance of similar multivariate models applied to other lignocellulosic systems (e.g., corn stover, poplar) where R^2^ values are commonly reported for predicting saccharification outcomes [33,34,36].

### 3.5. Prediction with the Built Model

**Figure 6:**
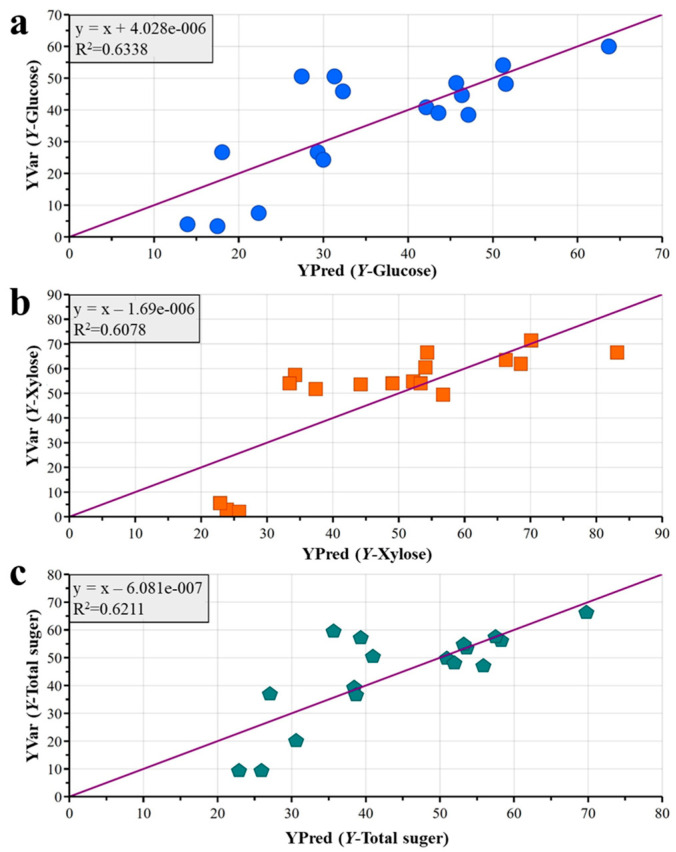
PLS enzymatic model verification of correlation between predicted values and test values ((**a**): *Y*-glucose, (**b**): *Y*-xylose, (**c**): *Y*-Total sugar).

To substantiate the predictive capabilities of the developed multivariate PLS model in the context of enzymatic hydrolysis, an additional 20 experimental datasets were procured. Figure 6 illustrates a comparison between the observed and predicted values for enzymatic saccharification results, encompassing *Y*-Glucose, *Y*-Xylose, and Y-Total sugar. The data points were symmetrically distributed around the line of equality, indicative of a good linear correlation. The R^2^ values of the regression line of *Y*-Glucose, *Y*-Xylose, and *Y*-Total sugar were 0.6338, 0.6078, and 0.6211, respectively. With all R^2^ values exceeding the threshold of 0.5, the PLS model suggests a relatively good fit for the enzymatic hydrolysis process and exhibits commendable predictive ability. In this case, with the known effectiveness of pretreatment and the set enzymatic hydrolysis conditions, the sugar yields can be predicted with this PLS model. This means that, to obtain relatively stable sugar yields, the conditions of enzymatic hydrolysis can be adjusted in advance based on the developed PLS model and the known effectiveness of pretreatment. Certainly, the developed PLS model needs to be periodically calibrated by including more datasets during real application, to keep the PLS model more robust.

## 4. Conclusions

This study demonstrated the utility of MDA in optimizing the enzymatic hydrolysis of alkali-pretreated wheat straw. PCA and PLS modeling of an extensive dataset identified cellulase dosage, PFI, DAV, and solid content as the most critical variables governing saccharification efficiency. A significant synergistic effect between cellulase and xylanase was quantified, revealing that optimal synergy occurs at moderate enzyme loadings. The developed PLS model exhibited strong predictive capability for sugar yields, validating its potential as a tool for process control and optimization. Despite the robust predictive performance of the developed PLS model, certain limitations should be acknowledged. The model was built and validated on data from a specific biomass (wheat straw). Its direct applicability to other feedstocks or vastly different pretreatment methods may require recalibration with new datasets. Furthermore, the model currently predicts sugar yields based on laboratory-scale hydrolysis parameters; scaling effects related to mass transfer, mixing, and heat distribution in industrial-scale reactors are not captured. Future work will focus on incorporating data from scaled-up processes and diverse biomass types to enhance the model’s generalizability and robustness for industrial biorefinery applications.

## Figures and Tables

**Table 1:** Degree of synergism under different enzymatic hydrolysis conditions.

Categories (Cellulase + Xylanase)	5 FPU/g + 40 IU/g		10 FPU/g + 40 IU/g		15 FPU/g + 40 IU/g	
NaOH dosage	7%	11%	7%	11%	7%	11%
*C*-Glucan	1.45	1.26	1.23	1.14	1.15	1.15
*C*-Xylan	1.04	1.02	1.02	0.96	0.93	0.90
*Y*-TS	1.13	1.12	1.06	1.03	1.02	1.01

Notes: *C*-Glucan, conversion rate of glucan; *C*-Xylan, conversion rate of xylan; *Y*-TS, final total sugar yield.

## Data Availability

The raw data supporting the conclusions of this article will be made available by the authors on request.

## Supplementary Materials

The following supporting information can be downloaded at: https://www.mdpi.com/article/10.3390/polym18020157/s1, Figure S1: Variable important plot for the whole PLS model (a) and the coefficient plots presenting the influence of independent variables on the specific Y variables. b: C-glucan, c: C-xylan, d: Y-glucose, e: Y-xylose, f: Y-Total sugar; Table S1: Pretreatment conditions of alkali pretreatment; Table S2: Chemical compositions of the raw and pretreated wheat straw; Table S3: Classification of key variables in enzymatic hydrolysis; Table S4: Components of PCA; Table S5: Components of PLS.

## Abbreviations

The following abbreviations are used in this manuscript:

MDA	multivariate data analysis
PCA	principal component analysis
PLS	partial least square
AQ	anthraquinone
SLS	sodium lignosulfonate
DS	degree of synergism
DAV	dye adsorption value
p[1]	the first principle component in PLA model
p[2]	the second principle component in PLA model
w*c[1]	the first principle component in PLS model
w*c[2]	the second principle component in PLS model
