# Supplementary material for: The Effect of Enzyme Synergism on Generation of Fermentable Sugars After Alkali Pretreatment of Wheat Straw, Assessed and Predicted Using Multivariate Analysis
Source: Polymers (Basel). 2026 Jan 7;18(2):157. doi: 10.3390/polym18020157 (PMC12845031; doi:10.3390/polym18020157)
Supplement: Supplementary file 1 [file polymers-18-00157-s001.zip › polymers-4060623-supplementary.pdf]

Supporting Information

The effect of enzyme synergism on generation of fermentable sugars after alkali pretreatment of wheat straw assessed and predicted using multivariate analysis

Yufa Gao [1, 2], Zhe Li [3], Zhibin Li [4], Xitao Luo [2, 3], Mohammad Ali Asadollahi [5], Safoora Mirmohamadsaghi [5], Guang Yu [2, *], Bin Li [2, 3, *]

[1] National Centre for Archaeology, Beijing 100013, China

[2] Qingdao New Energy Shandong Laboratory, System Integration Engineering Center, Qingdao Institute of Bioenergy and Bioprocess Technology, Chinese Academy of Sciences, Qingdao 266101, China

[3] Weifang Engineering Research Center of Functional Sugar and Polvols, Gaomi Tongliao Sugar Co., Ltd., Weifang 261599, China

[4] China National Pulp and Paper Research Institute Co., Ltd., Beijing 100102, China

[5] Department of Biotechnology, Faculty of Biological Science and Technology, University of Isfahan, Isfahan, 81746-73441, Iran

Corresponding authors' E-mail addresses: yuguang@qibebt.ac.cn (G. Yu); libin@qibebt.ac.cn (B. Li)

Table S1. Pretreatment conditions of alkali pretreatment.

NaOH (%)	SLS (%)	AQ (%)	Liquid to solid	Temperature (°C)	Time (min)
7	—	—	6:1	120	40
8	—	—	6:1	120	40
9	—	—	6:1	120	40
11	—	—	6:1	120	40
13	—	—	6:1	120	40
8	2	0.1	6:1	120	40
9	2	0.2	4:1	90	90
11	2	—	6:1	120	40
12	2	0.1	6:1	120	40
12	2	0.2	4:1	90	90

Note: SLS, sodium lignosulfonate.

Table S2. Chemical compositions of the raw and pretreated wheat straw.

Samples	Glucan (%)	Xylan (%)	Lignin (%)	Extractives (%)	Ash (%)	Sum (%)
Raw materials	31.97±0.45	13.99±0.52	20.64±0.23	20.16±0.48	11.23±0.07	97.99
7%NaOH	45.89±0.52	18.19±0.46	13.94±0.67	12.05±0.94	7.09±0.15	97.16
8%NaOH	49.78±0.15	19.77±0.28	11.43±0.94	10.59±0.85	6.04±0.24	97.61
9%NaOH	54.27±0.47	23.02±0.39	9.23±0.25	8.98±0.92	3.26±0.35	98.76
11%NaOH	52.73±0.58	22.32±0.87	7.95±0.17	12.13±0.36	3.76±0.27	98.89
13%NaOH	53.41±0.69	22.38±0.64	7.57±0.22	9.73±0.35	3.92±0.39	97.01
8%NaOH+ 2%SLS+0.1%AQ	53.78±0.76	23.08±0.03	8.25±0.38	10.14±0.71	3.85±0.36	99.1
9%NaOH+ 2%SLS+0.2%AQ	63.82±0.95	23.71±0.29	3.41±0.97	5.27±0.58	3.60±0.71	99.81
11%NaOH+ 2%SLS	50.19±0.81	20.69±0.45	8.91±0.71	13.27±0.23	4.91±0.86	97.97
12%NaOH+ 2%SLS+0.1%AQ	56.26±0.69	23.72±0.57	6.39±0.82	10.13±0.44	3.17±0.09	99.67
12%NaOH+ 2%SLS+0.2%AQ	62.27±0.35	24.16±0.61	3.32±0.63	5.26±0.62	3.39±0.54	98.40

Note: SLS, sodium lignosulfonate; AQ, anthraquinone.

Table S3. Classification of key variables in enzymatic hydrolysis.

Category	Name of variable	Value range of variable
Compositions of the pretreated wheat straw	Glucan	45.89-63.82 %
	Xylan	18.19-24.16%
	Extractives	5.26-13.27%
	Lignin	3.32-13.94%
	Ash	3.17-7.09%
The parameters of enzymatic hydrolysis conditions	DAV	336.49-613.53 mg/g
	PFI	0-8000 r
	Cellulase	0-20 FPU/g
	Xylanase	0-80 IU/g
	Solid content	1-10%
	Time	12-72 h
	pH	4-5.4
	Temperature	40-60 ^{o}C
The evaluation of enzymatic effect	*C*-Glucan	0.00-89.96%
	C-Xylan	1.38-99.93%
	Y-Glucose	0.02-81.31%
	Y-Xylose	1.21-80.96%
	Y-Total Sugar (*Y*-TS)	6.39-83.31%

Note: DAV, dye adsorption value; PFI, revolution number of PFI refining; Cellulase, cellulase dosage; Xylanase, xylanase dosage; *C*-Glucan, conversion rate of glucan; *C*-Xylan, conversion rate of xylan; *Y*-Glucose, yield of glucose; *Y*-Xylose, yield of xylose; *Y*-Total Sugar (*Y*-TS), yield of total sugar.

Table S4. Components of PCA.

Categories	A	R^2X	R^2X(cum)	Q^2	Q^2(cum)	Significance
PCA	1	0.343	0.343	0.295	0.295	R1
	2	0.196	0.539	0.152	0.447	R1
	3	0.107	0.646	0.083	0.530	R1

Table S5. Components of PLS.

Categories	A	R^2X	R^2X(cum)	R^2Y	R^2Y(cum)	Q^2	Q^2(cum)	Significance
PLS	1	0.192	0.192	0.43	0.43	0.403	0.403	R1
	2	0.144	0.336	0.205	0.635	0.301	0.704	R1
	3	0.232	0.568	0.045	0.680	0.084	0.788	R1

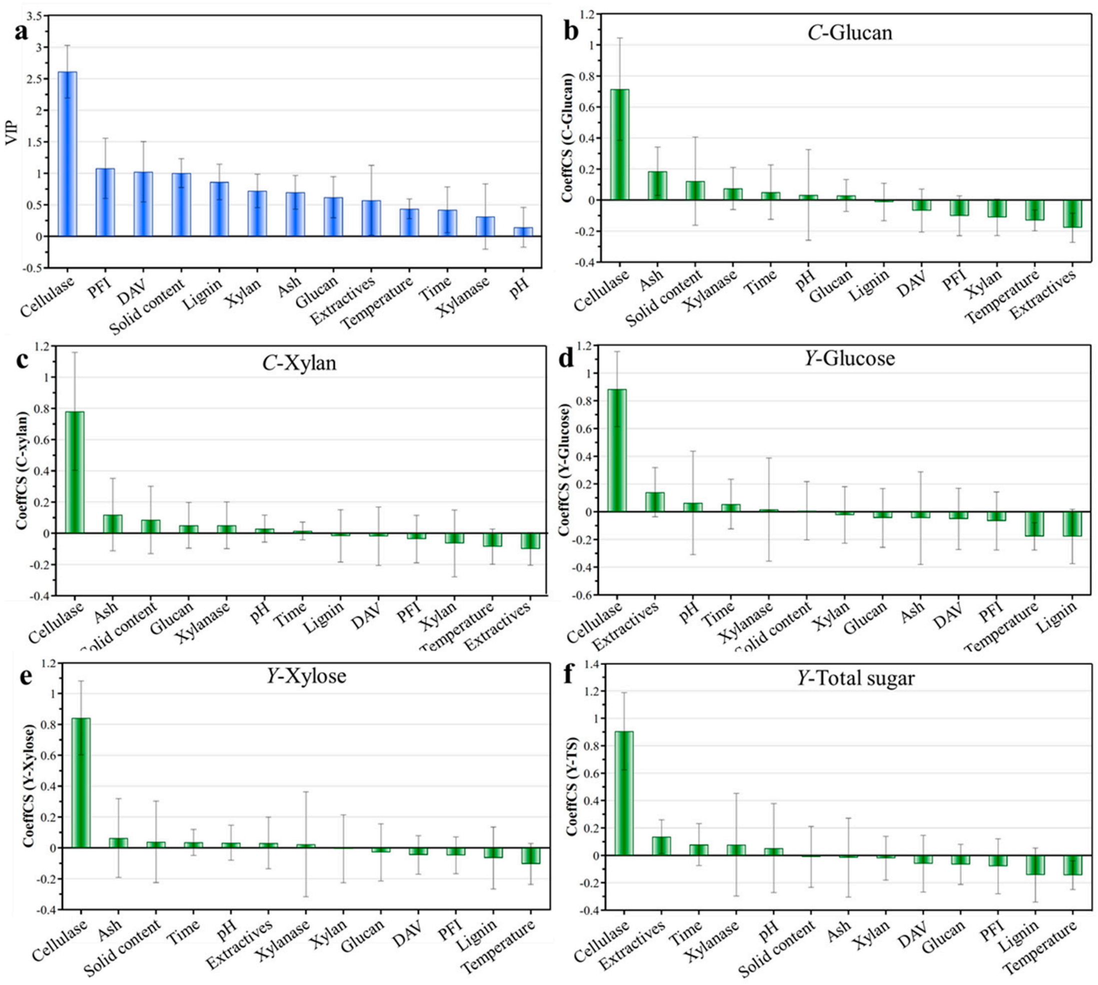

Figure S1. Variable important plot for the whole PLS model (a) and the coefficient plots presenting the influence of independent variables on the specific Y variables. b: *C*-glucan, c: *C*-xylan, d: *Y*-glucose, e: *Y*-xylose, f: *Y*-Total sugar
